# Fermented Red Ginseng Restores Age-Associated Insulin Homeostasis and Gut Microbiome Balance in Mice

**DOI:** 10.3390/biology15030211

**Published:** 2026-01-23

**Authors:** Da-Yeon Lee, Jing Liu, Gopal Lamichhane, Ashton Swayze, Guolong Zhang, Tae Young Kim, Josephine M. Egan, Yoo Kim

**Affiliations:** 1 Department of Nutritional Sciences, Oklahoma State University, Stillwater, OK 74078, USA; dayeon.lee@okstate.edu (D.-Y.L.); gopal.lamichhane@okstate.edu (G.L.); ashton.swayze@okstate.edu (A.S.); 2 Department of Animal and Food Sciences, Oklahoma State University, Stillwater, OK 74078, USA; jing.liu12@okstate.edu (J.L.); glenn.zhang@okstate.edu (G.Z.); 3 BTC Corporation, Gwacheon-si 13840, Gyeonggi-do, Republic of Korea; tykim@btcbio.com; 4 Laboratory of Clinical Investigation, National Institute on Aging, Baltimore, MD 21224, USA; eganj@grc.nia.nih.gov

**Keywords:** fermented red ginseng, aging, insulin homeostasis, inflammation, gut microbiome

## Abstract

Biological aging disrupts liver–gut intercommunication, resulting in the development of insulin resistance and type 2 diabetes, coupled with the imbalance of gut microbiome composition known as gut dysbiosis. Fermented red ginseng (FRG) is a renowned functional food substance showing its notable anti-inflammatory and anti-diabetic effects owing to its unique bioactive compounds known as ginsenosides. However, whether FRG could impact biological aging and age-related metabolic dysfunction is still unclear. The current study aimed to determine the health benefits of FRG in improving age-associated impaired insulin homeostasis and gut dysbiosis in 19-month-old male mice. Mice were fed with a normal chow diet (NCD) or NCD with FRG (300 mg/kg) for 14 weeks. FRG supplementation significantly improved insulin homeostasis by activating the hepatic protein kinase B (AKT) and proline-rich AKT substrate of 40 kDa (PRAS40). We also observed suppressed mRNA expression of proinflammatory cytokines and diminished inflammatory infiltrates in the liver of FRG-fed mice compared with NCD-only controls. Furthermore, alongside a decreased ratio of Firmicutes to Bacteroidetes, FRG administration enriched beneficial genera, including *Muribaculaceae*, *Borkfalkiaceae*, *Parasutterella*, and *Clostridia vadin BB60 group*, whereas FRG reduced the abundance of *Erysipelotrichaceae* and *Dubosiella* at the genus level. In summary, we suggest that FRG can be a potential anti-aging dietary supplement to manage age-driven dysregulation of insulin homeostasis and gut microbiota composition.

## Simple Summary

The goal of this study was to investigate the potential role of fermented red ginseng (FRG) in mitigating age-associated disruptions in insulin homeostasis and gut microbiome composition using 19-month-old aged male mice. Together with the improved insulin homeostasis, FRG supplementation activated insulin-related signaling pathways and suppressed proinflammatory cytokines in the liver. In addition, we found that FRG showed a pronounced influence on the microbial community by restoring the balance between commensal and opportunistic bacteria that is compromised during aging. Taken together, our findings highlight that FRG positively impacts the aging process by preserving insulin homeostasis and gut microbiota compositional equilibrium.

## 1. Introduction

Aging is a progressive biological and functional deterioration that leads to the increased risk of a wide range of chronic human pathologies, such as metabolic syndromes, cardiovascular diseases, neurodegenerative diseases, and cancer [1]. Among these pathophysiological conditions, elderly populations are becoming more susceptible to type 2 diabetes (T2D), so-called geriatric diabetes, due to age-driven insulin resistance or hyperinsulinemia [2,3]. One of the major metabolic organs compromised by the aging process is the liver, and this phenomenon is caused by the dysregulation of insulin-related signaling molecules, including protein kinase B (PKB; AKT) and proline-rich AKT substrate of 40 kDa (PRAS40) [4,5,6]. Aging also disrupts the equilibrium of microbial community, leading to dysbiosis, and recent studies have reported that age-associated dysbiosis contributes to the development of insulin resistance and T2D [7,8,9]. Hence, targeting the bidirectional communication between the liver and gut in the context of aging may serve as a strategic approach to managing geriatric diabetes and dysbiosis [10].

In recent years, dietary and nutritional interventions have gained increasing attention as potential therapeutic applications to mitigate aging and age-related diseases [11]. Ginseng (*Panax ginseng* C.A. Meyer), the root of a perennial umbelliferous plant belonging to the genus *Panax* and the family *Araliaceae*, has been traditionally used as an herbal medicine in East Asian countries for thousands of years [12]. Raw and fresh ginseng can be processed into white, red, or black ginseng through repetitive steaming and drying processes up to nine cycles to enhance its bioactive components [13,14]. Produced through a one-time thermal process, red ginseng (RG) is one of the most widely studied natural food substances for its pharmacological benefits, especially exerting anti-aging properties by attenuating age-related diabetes, inflammation, cognitive decline, and sarcopenia [15,16,17,18,19]. Besides conventional thermal procedure, fermentation using microorganisms is an effective method not only to increase bioactive compounds (mainly ginseng-specific saponins called ginsenosides) and microbial metabolites in ginseng products but also to augment their health benefits [20]. Specific bacterial species have been used for the fermentation of RG, including *Lactobacillus* sp., *Bifidobacterium*, and *Saccharomyces cerevisiae*, to produce minor ginsenosides (e.g., compound K, ginsenosides Rg3, and Rh2) [21]. Previous studies have reported the enhanced health benefits of fermented red ginseng (FRG) over RG on ameliorating diabetes, hypertension, ischemic brain injury, depression, and dysbiosis-induced ulcerative colitis [22,23,24,25]. In terms of age-associated conditions, FRG has shown protective effects against muscle atrophy and skin aging [26,27]. However, whether FRG can modulate age-driven diabetes or gut dysbiosis under naturally aged conditions is not fully understood.

In the current study, we hypothesized that FRG could alleviate age-associated dysregulation of insulin homeostasis and modulate gut microbiome composition. To test this, we examined the regulatory effects of FRG fermented by *Lactobacillus brevis* M2 using a naturally aged mouse model.

## 2. Materials and Methods

### 2.1. Preparation of FRG Extracts

*Lactobacillus brevis* M2 (KCTC 11390BP) was used for FRG preparation by isolating from colonies cultured on Difco™ De Man–Rogosa–Sharpe (MRS) agar (Benton, Dickinson and Company, Franklin Lakes, NJ, USA) for 48 h at 37 °C, with the inoculation of the commercial FRG products. A total of 2.5 g of RG powder (Jungwon Ginseng, Geumsan-gun, Republic of Korea) was dissolved in 50 mL of 50 mM phosphate-buffered saline (pH 5.0) and then sterilized. The precultured *L. brevis* M2 strain was inoculated into the sterilized RG suspension at 2%, and then incubated at 37 °C for 5 days with mild shaking.

### 2.2. Compositional Analysis of FRG

For high-performance liquid chromatography (HPLC) analysis, 1 g of FRG was extracted with 70% ethanol at 80 °C twice via a shaking incubator. The obtained extracts were filtered and evaporated using a rotary evaporator under vacuum, followed by dissolving 50 mL of distilled water. Next, 1 mL of dissolved extract was mixed with 2 mL of diethyl ether and centrifuged at 1500 rpm for 10 min. Then, the diethyl ether layer was removed, and 1.5 mL of water-saturated n-butanol was added. After repeating the process three times, the butanol extract was washed with 1.5 mL of distilled water twice to remove the water-soluble impurities. The remaining butanolic solution was dried by the nitrogen blowdown evaporation at 40 °C, and 0.5 mL of methanol was added for HPLC analysis. All samples were filtered through 0.45 µm membrane syringe filters.

The HPLC system was equipped with SP930D HPLC Column Solvent Delivery Pump, SDV50A Vacuum Degasser and Valve module, CTS30 Column Oven (Young Lin Co. Ltd., Anyang-si, Gyeonggi-do, Republic of Korea), and ELSD detector (Alltech Associates, Inc., Deerfield, IL, USA). The amounts of ginsenosides in FRG were analyzed by a Prevail™ Carbohydrate ES Column (4.6 × 250 mm, 5 µm; Alltech Associates, Inc.) at 35 °C of column oven temperature. The mixture of mobile phase A (acetonitrile–water–isopropyl alcohol = 80:5:15) and B (acetonitrile–water–isopropyl alcohol = 67:21:12) was used at a flow rate of 0.8 mL/min with 20 μL of sample injection volume. The gradient of mobile phases was varied as follows: 0 min, 20% B; 28 min, 85% B; 35 min, 80% B; 45 min, 75% B; 50 min, 90% B; 51 min, 100% B; 57 min, 25% B; and 58–65 min, 10% B. The HPLC-grade standards of ginsenosides were purchased from AMBO Institute (Daejeon, Republic of Korea).

### 2.3. Animals

All animal experimental procedures were approved by the Institutional Animal Care and Use Committee (IACUC) of Oklahoma State University and performed at the Oklahoma State University Animal Resource Facility, accredited by the American Association for Accreditation of Laboratory Animal Care (AAALAC). The 19-month-old male C57BL/6 mice were obtained from the National Institute on Aging (NIA) Aged Rodent Colony at Charles River Laboratories (Frederick, MD, USA). Upon arrival, mice were acclimated for one week, followed by the baseline measurements. Mice were then randomly assigned into two groups: a normal chow diet (NCD) or NCD with 300 mg/kg of FRG (NCD + FRG) (n = 9 per group). The dosage of FRG was determined according to the previous study [28]. Mice were allowed access to diet and water ad libitum for 14 weeks. FRG-customized diets were formulated by Dyets, Inc. (Bethlehem, PA, USA) (Supplementary Table S1). Body weight and cumulative food intake were carefully monitored once a week throughout the study. After 14 weeks, mice were sacrificed via cervical dislocation, following blood collection through retro-orbital bleeding, and biological samples were collected for further analyses.

### 2.4. Glucose and Insulin Tolerance Tests

The glucose tolerance test (GTT) was performed by measuring blood glucose levels from the tail vein of 16 h-fasted mice at 0, 15, 30, 60, 90, and 120 min after intraperitoneal (IP) injection of 2 g/kg glucose using a 50% (*w*/*w*) glucose solution (Alpha Teknova Inc., Hollister, CA, USA). For the insulin tolerance test (ITT), fasted blood glucose levels were measured from the tail vein at 0, 15, 30, 60, 90, and 120 min following IP injection of 0.5 IU/kg recombinant human insulin (Novo Nordisk Inc., Plainsboro, NJ, USA). Blood glucose levels were assessed using the Contour^®^ Next EZ Blood Glucose Monitoring System (Ascensia Diabetes Care, Parsippany, NJ, USA).

### 2.5. Serum Insulin Level Measurement

Mouse blood samples were collected from the retro-orbital sinus prior to termination following a 6 h fasting condition. Blood samples were centrifuged at 3000 rpm for 20 min at 4 °C to collect serum. Serum insulin levels were measured using the Ultra Sensitive Mouse Insulin ELISA Kit (Crystal Chem, Elk Grove Village, IL, USA) following the manufacturer’s instructions. The homeostasis model assessment of insulin resistance (HOMA-IR) was calculated by the following equation: fasting blood glucose (mmol/L) × fasting insulin (µIU/mL)/22.5.

### 2.6. Immunoblotting Assay

Mouse liver tissues were homogenized with T-PER™ Tissue Protein Extraction Reagent (Thermo Fisher Scientific, Waltham, MA, USA) containing PhosSTOP™ Phosphatase Inhibitor and cOmplete™ Mini Protease Inhibitor Cocktail (Roche; Sigma-Aldrich, St. Louis, MO, USA). The concentrations of extracted proteins were normalized by the bicinchoninic acid (BCA) assay kit (Thermo Fisher Scientific). The normalized protein samples were loaded and resolved in sodium dodecyl sulfate–polyacrylamide gel electrophoresis (SDS-PAGE) under reducing conditions, followed by transfer to polyvinylidene fluoride (PVDF) membranes. Membranes were blocked with a commercial blocking buffer (Intercept^®^; LI-COR Biotechnology, Lincoln, NE, USA) for 1 h at room temperature and subsequently incubated at 4 °C overnight with the following primary antibodies: phosphorylated AKT (p-AKT)^Ser473^, AKT (pan), p-PRAS40, PRAS40, and β-actin (Cell Signaling Technology, Inc., Danvers, MA, USA). After incubation, membranes were washed three times for 10 min each with tris-buffered saline with Tween 20 (TBS-T), and then incubated with anti-rabbit IgG secondary antibody (Cell Signaling Technology) diluted in 5% non-fat skim milk in TBS-T for 1 h at room temperature. Following three-time TBS-T washes, immunoblots were developed using a chemiluminescence assay system (Thermo Fisher Scientific) and visualized on autoradiographic X-ray films (Thomas Scientific, Swedesboro, NJ, USA). The original immunoblots are presented in Figure S1.

### 2.7. RNA Sequencing Analysis

Total RNA was extracted from mouse liver tissues stored in RNAlater (Sigma-Aldrich) via a Qiagen RNeasy Mini Kit (Qiagen, Hilden, Germany). RNA samples were subjected to quality control in terms of their purities and integrities, and were subsequently processed for library preparation and mRNA sequencing (Novogene Corporation, Ltd., University of California, Davis, CA, USA). An Illumina NovaSeq 6000 platform (Illumina, Inc., San Diego, CA, USA) was employed with a read length of 2 × 150 base pairs. Differentially expressed genes (DEGs) were identified based on transcriptome analysis results (*p* < 0.05; |log2FoldChange| > 0). Selected DEGs were then used for biological function and pathway analyses through the Ingenuity Pathway Analysis (IPA) software (Qiagen).

### 2.8. Real-Time Reverse Transcription-Polymerase Chain Reaction (RT-PCR)

Total RNA was isolated from frozen liver tissues using TRIzol Reagent (Invitrogen; Thermo Fisher Scientific), and reverse transcribed into cDNA with the iScript™ cDNA Synthesis Kit (Bio-Rad Laboratories, Hercules, CA, USA). mRNA expression was evaluated using SYBR Green PCR Master Mix (Applied Biosystems; Thermo Fisher Scientific) on a CFX Opus 384 Real-Time PCR System (Bio-Rad Laboratories) under the following thermal cycling conditions: 50 °C for 2 min, 95 °C for 10 min, followed by 39 cycles at 95 °C for 15 s and 60 °C for 1 min. Tumor necrosis factor alpha (TNF-α), interleukin (IL)-1β, IL-6, myeloid differentiation primary response 88 (Myd88), chemokine (C-X-C motif) ligand 2 (Cxcl2), plasminogen activator inhibitor-1 (Pai-1), monocyte chemoattractant protein 1 (Mcp1), matrix metalloproteinase 12 (Mmp12), and tissue inhibitor of metalloproteinases 1 (TIMP1) were analyzed. Fluorescence cycle threshold (Ct) values were normalized to 18S ribosomal RNA. Primer sequences used in this study are listed in Supplementary Table S2.

### 2.9. Histological Analysis

Liver tissues collected from each group of mice were fixed overnight in freshly prepared 4% paraformaldehyde. Formalin-fixed liver tissues were dehydrated through an ethanol gradient (70, 80, 95, and 100%) and toluene using a Shandon Citadel 1000 Tissue Processor (Thermo Fisher Scientific), followed by paraffin embedding. Samples were sectioned at a thickness of 5 µm by a rotary microtome (RM2165; Leica Biosystems, Nussloch, Germany). After de-paraffinization and rehydration, sections were stained with hematoxylin and eosin (H&E). Structural changes in the liver were visualized at 20× magnification using a Keyence BZ-X710 microscope (Itasca, IL, USA). Liver inflammation scores were assessed under the NAFLD activity score (NAS) criteria: 0 for no inflammation; 1 for mild inflammation; 2 for moderate inflammation; 3 for severe inflammation (n = 4 per group).

### 2.10. 16S rRNA Sequencing Analysis

Genomic DNA was isolated from the mouse fecal samples collected before termination using the QIAmp Fast DNA Stool Mini Kit (Qiagen), followed by paired-end sequencing of the V3–V4 region of the 16S ribosomal RNA (rRNA) gene. An Illumina NovaSeq 6000 platform (Illumina, Inc.; Novogene Corporation) was utilized to generate the sequencing library, and sequencing reads were processed by QIIME 2 (v. 2023.07). Amplicon sequence variants (ASVs) were classified using the Ribosomal Database Project (RDP) 16S rRNA training set (v. 18) and the Bayesian classifier [29]. Taxa with a bootstrap confidence of <80% were assigned to the last confidently classified taxonomic level, followed by ‘_unclassified.’ The top 30 ASVs and all differentially enriched ASVs were further validated and reclassified, if necessary, based on the updated EzBioCloud 16S database (v. 2023.08.23). Differential enrichment of bacterial ASVs between each group was determined using linear discriminant analysis (LDA) effect size (LEfSe) with all-against-all multiclass analysis (*p* < 0.05) and a logarithmic LDA score threshold of >2.5.

### 2.11. Statistical Analysis

All experimental data were analyzed by GraphPad Prism 10 (GraphPad Software, San Diego, CA, USA). An ordinary two-way repeated-measure analysis of variance (ANOVA) was applied to evaluate body weight gains, cumulative food intake, ITT, and GTT results, followed by Tukey’s multiple comparison tests. Student’s *t*-test was applied for the assessment of the area under the curve (AUC), fasting blood glucose levels, serum insulin levels, HOMA-IR, and immunoblot band intensity after an outlier test (α = 0.05). All quantitative data are expressed as mean ± standard deviation (SD).

## 3. Results

### 3.1. FRG Maintains Insulin Homeostasis by Increasing Insulin Sensitivity in Aged Mice

**Figure 1:**
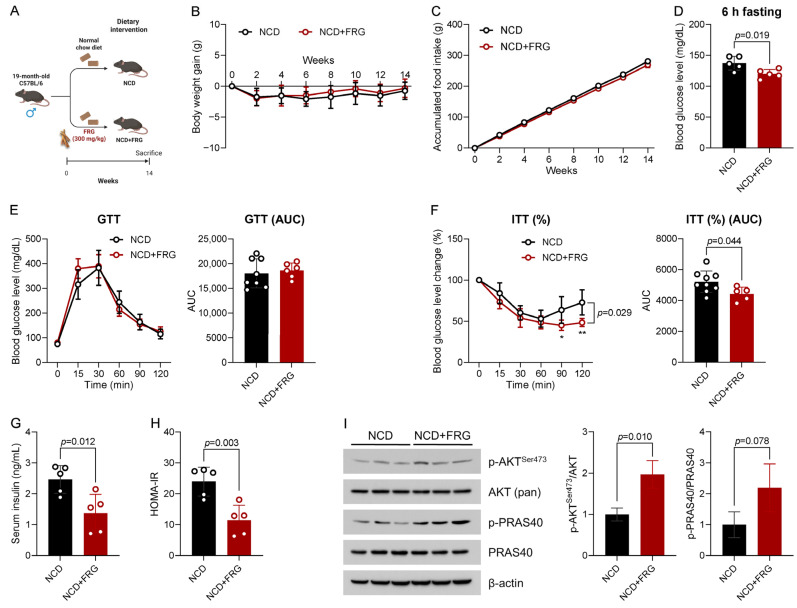
FRG improves insulin homeostasis by activating hepatic AKT signaling in aged mice. (**A**) Experimental design. The 19-month-old C57BL/6 male mice were fed a normal chow diet (NCD) or an NCD with 300 mg/kg FRG (NCD + FRG) for 14 weeks (n = 9 per group). (**B**) Body weight gain (g) and (**C**) accumulated food intake (g) were monitored every two weeks. (**D**) The 6 h-fasted blood glucose level (mg/dL) was measured before euthanasia. (**E**) Glucose tolerance test (GTT; 2 g/kg) (**left**) on week 12 and the calculation of area under the curve (AUC) (**right**). (**F**) Insulin tolerance test (ITT; 0.5 IU/kg) (**left**) on week 11 and the calculation of AUC (**right**). (**G**) Serum insulin level (ng/mL). (**H**) Homeostasis model assessment of insulin resistance (HOMA-IR). (**I**) Representative immunoblots (**left**) and quantification (**right**) of p-AKT^Ser473^, AKT (pan), p-PRAS40, and PRAS40 in liver tissue lysates, normalized to β-actin. Data are presented as mean ± standard deviation (SD) (* *p* < 0.05, ** *p* < 0.01).

We previously reported that dietary RG was beneficial to improve several age-related symptoms, including impaired insulin homeostasis, in naturally aged mice [16]. In this study, we sought to investigate whether fermented RG, FRG, could also exert health benefits under aged conditions. To address this question, we conducted a 14-week ad libitum FRG intervention in 19-month-old male mice (Figure 1A). FRG supplementation maintained body weight (Figure 1B) and did not affect cumulative food intake (Figure 1C) throughout the intervention period. Subsequently, we assessed the 6 h-fasted blood glucose level prior to termination of the study, and we found that FRG lowered fasting blood glucose level (Figure 1D). We also observed no significant differences in GTT responses between the NCD and NCD + FRG groups (Figure 1E). Intriguingly, ITT results demonstrated that FRG supplementation significantly improved insulin sensitivity in FRG-fed mice (Figure 1F). Supporting the ITT results, we confirmed that FRG markedly decreased circulating insulin levels (Figure 1G) and HOMA-IR (Figure 1H), compared with the NCD-only-fed group.

In terms of insulin homeostasis, AKT is highlighted due to its regulatory role in insulin signaling, yet its activity decreases in insulin-sensitive organs, including the liver, during aging [30]. Thus, we further examined the effect of FRG supplementation on AKT signaling. We observed that FRG treatment remarkably increased AKT phosphorylation at the serine 473 site. This was consistent with an accompanying increase in phosphorylation of PRAS40, a major downstream substrate of AKT and a key component of the mechanistic target of rapamycin complex 1 (mTORC1) (Figure 1I). Taken together, these results elucidate that FRG improves insulin homeostasis in aged mice by activating hepatic AKT and its downstream signaling molecule.

### 3.2. FRG Downregulates Inflammation-Related Hepatic Gene Expressions in Aged Mice

**Figure 2:**
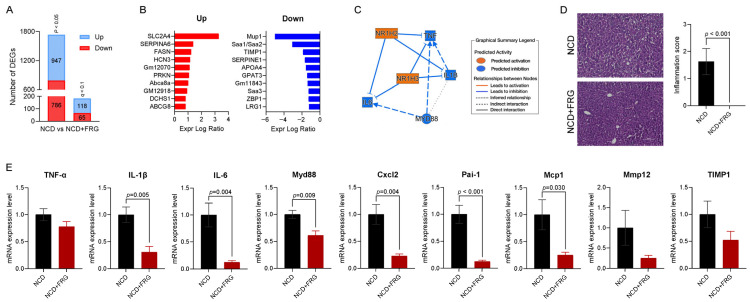
FRG regulates inflammation-related hepatic gene expression in aged mice. (**A**) Numbers of differentially expressed genes (DEGs) identified by *p*-values (*p* < 0.05) and False Discovery Rates (FDR)-defined q-value cut-offs (*q* < 0.1) in the comparison between NCD and NCD + FRG groups. (**B**) Expression log ratios of significantly upregulated (**left**) and downregulated (**right**) genes (*p* < 0.05). (**C**) Graphical summary of molecular activity prediction analysis generated using Ingenuity Pathway Analysis (IPA). (**D**) Representative images from hematoxylin and eosin (H&E)-stained liver sections (scale bar = 100 µm) (**left**) and inflammation scores (**right**). (**E**) Hepatic mRNA expression levels of proinflammatory markers. Data are presented as mean ± SD.

Given the potential of FRG to positively impact insulin homeostasis in aged mice, we next sought to investigate the possible mechanisms that might underlie this effect. To this end, we performed hepatic transcriptome analysis using RNA sequencing (RNA-seq) in NCD and NCD + FRG-fed mice. As shown in Figure 2A, FRG markedly altered hepatic gene expression profiles compared with non-FRG-treated mice. A total of 947 genes were significantly upregulated and 786 genes were downregulated in the NCD + FRG group compared to the NCD group (Figure 2A). DEGs selected from RNA-seq were subjected to further functional and pathway analysis using IPA software. IPA identified the top 10 upregulated and top 10 downregulated DEGs with the greatest expression changes in response to FRG (Figure 2B). Based on these DEGs data, molecular activity prediction analysis summarized that proinflammatory cytokines, including TNF-α, IL-1β, IL-6, and Myd88, were predicted to be inhibited by FRG supplementation (Figure 2C). Histological analysis also confirmed that livers from FRG-fed mice showed minimal inflammatory infiltrates compared with the NCD group (Figure 2D). To validate these findings, we quantified mRNA expression levels of these four cytokines, along with five additional inflammatory mediators, Cxcl2, Pai-1, Mcp1, Mmp12, and TIMP1, also known as senescence-associated secretory phenotypes (SASPs). Consistent with the transcriptomic predictions, FRG suppressed the expression of all nine genes (Figure 2E). Overall, these results suggest that FRG exerts a modest but meaningful anti-inflammatory effect in the liver, contributing to the improved insulin homeostasis observed in aged mice under normal diet conditions.

### 3.3. FRG Positively Alters Gut Microbiome Composition in Aged Mice

**Figure 3:**
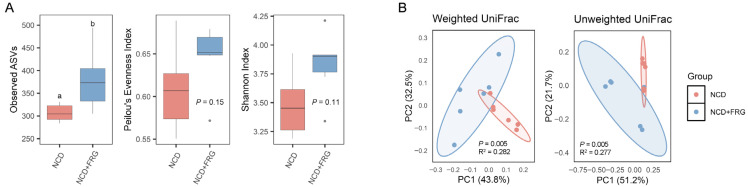
FRG positively alters gut microbiota diversity in aged mice. (**A**) Alpha diversity represented by observed amplicon sequence variants (ASVs) (**left**), Peilou’s evenness index (**middle**), and Shannon index (**right**). Different letters indicate statistically significant differences (*p* < 0.05). (**B**) Beta diversity assessed using principal coordinate analysis (PCoA) based on weighted (**left**) and unweighted (**right**) UniFrac distances. Statistical significance was determined by PERMANOVA using 999 permutations (n = 5–6 per group).

In addition to geriatric diabetes, age-associated chronic inflammation adversely affects the structure and composition of the gut microbiome, leading to gut dysbiosis [30,31]. Given that FRG improved insulin homeostasis and reduced hepatic inflammation markers, we next examined whether FRG also modulates gut microbial equilibrium in aged mice. To assess this, we analyzed gut microbiome composition in NCD- and NCD + FRG-fed mice. Our observation discovered that FRG supplementation significantly increased gut microbial alpha diversity, reflected by a higher number of observed ASVs (*p* < 0.05), although Pielou’s evenness index and Shannon index did not reach statistical significance (Figure 3A). Moreover, principal coordinate analysis (PCoA) based on UniFrac distances revealed distinct microbial clustering in the NCD + FRG group, both quantitatively (R^2^ = 0.282, *p* = 0.005; weighted UniFrac) and qualitatively (R^2^ = 0.277, *p* = 0.005, unweighted UniFrac), compared to the NCD group (Figure 3B). These results indicate that FRG significantly alters microbial diversity and community structure in aged mice.

**Figure 4:**
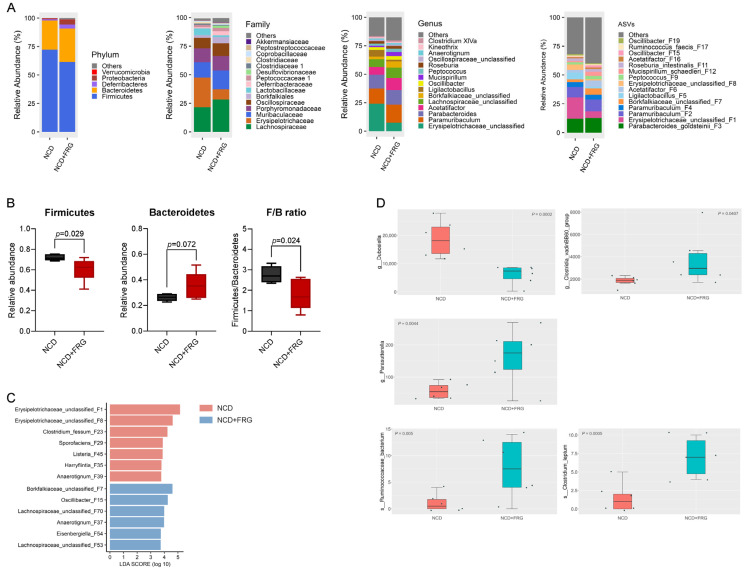
FRG alters gut microbiome composition in aged mice. (**A**) Relative abundance of gut microbiota at the phylum, family, genus, and ASV levels. (**B**) Relative abundance of Firmicutes (**left**) and Bacteroidetes (**middle**), and the ratio of Firmicutes to Bacteroidetes (F/B) (**right**). (**C**) Differential enrichment of bacterial ASVs identified by linear discriminant analysis (LDA) effect size (LEfSe) analysis (*p* < 0.05, LDA > 2.5). (**D**) MetaStat analysis expressing taxa with significant intergroup variation between NCD and NCD + FRG groups at the genus (g_) and species (s_) levels.

Compositional analysis further confirmed major shifts in predominant phyla, including Firmicutes, Bacteroidetes, Deferribacteres, and Proteobacteria (Figure 4A). The top 15 families, 15 genera, and 15 ASVs enriched in each group were identified, as shown in Figure 4A. In particular, FRG significantly reduced the abundance of Firmicutes and modestly increased Bacteroidetes, resulting in a significantly decreased ratio of Firmicutes to Bacteroidetes (F/B ratio) (Figure 4B). This change was largely driven by a decline in the Firmicutes family *Erysipelotrichaceae*, together with increased abundance of *Muribaculaceae*, belonging to Bacteroidetes, and enhancement of the family *Borkfalkiales*. Consistent with these findings, similar patterns were observed at both the genus and ASV levels, including reduced relative abundances of *Erysipelotrichaceae_unclassified* (F1 and F8), and elevated abundance of *Borkfalkiaceae_unclassified* ASVs in the NCD + FRG group (Figure 4A). These observations aligned with LEfSe analysis, which additionally identified two *Lachnospiraceae_unclassified* ASVs (F53 and F70) enriched in the NCD + FRG group (LDA > 2.5) (Figure 4C). We also identified several taxa with significant differences between groups, including *Dubosiella*, *Clostridia vadin BB60 group*, *Parasutterella* (genus), *Ruminococcus faecis*, and *Clostridium leptum* (ASVs) (Figure 4D). Collectively, these results support that FRG positively impacts the gut microbiota of aged mice by reshaping both microbial structure and taxonomic composition.

## 4. Discussion

Aging is a complex, multifactorial process that increases the prevalence of various physical and metabolic dysfunctions [32]. In particular, this time-dependent biological decline can aggravate dysregulation of insulin-related signaling pathways in metabolic organs, chronic inflammation, and disruption of the intestinal equilibrium, widely considered as hallmarks of aging [7]. It is essential to understand the morphological and physiological alterations in key metabolic organs such as the liver and gut, as well as their bidirectional crosstalk, to maintain homeostasis in advanced age [31]. Decades of research in geroscience suggest that dietary interventions, including gerotherapeutics, can improve health span by selectively targeting specific hallmarks of aging and their molecular mechanisms, thereby preventing or ameliorating age-associated phenotypes and chronic diseases [33,34]. Natural products with a long history of use in human disease management have gained considerable attention as alternative strategies to mitigate the adverse effects often associated with synthetic gerotherapeutic drugs and chemicals [35,36].

In this study, we aimed to investigate whether FRG, fermented by the *L. brevis* M2 strain, moderates the age-driven pathophysiological conditions, particularly focusing on liver and gut health and their underlying mechanisms. We first confirmed that FRG contained higher levels of rare ginsenosides in FRG, including compound K, ginsenosides Rh2, Rg3, Rg5, and Rk1, more than non-fermented RG (Supplementary Table S3). For the animal study, we used a taste-masked FRG-customized diet by γ-cyclodextrin encapsulation to facilitate consistent intake by the mice. Although we observed body weight loss during the first two weeks of intervention, the FRG-fed group exhibited the same initial weight-loss pattern as the control group, and no differences in food intake were detected between groups. After this brief acclimation period to the FRG-specialized diet, mice recovered their body weights and maintained stable growth trajectories for the remainder of the study.

There has been a growing awareness of diabetes mellitus in the elderly population, as diabetes can exacerbate other age-associated chronic diseases [3]. AKT signaling is required for insulin and glucose homeostasis, but aging decreases AKT phosphorylation in the liver, leading to lipid accumulation, hyperinsulinemia, and the onset of diabetes [37]. Age-related chronic inflammation, so-called inflammaging, exacerbates ATK dysfunction and increases the secretion of proinflammatory cytokines (e.g., TNF-α, IL-1β, and IL-6) [30,38]. In this study, together with improved insulin homeostasis and plasma insulin clearance, we observed that FRG supplementation enhanced hepatic AKT phosphorylation at the serine 473 site and increased PRAS40 activation in naturally aged mice. In addition, FRG attenuated inflammatory infiltration and reduced the mRNA expression of proinflammatory cytokines and SASPs in the liver. These effects are likely attributed, in part, to compound K, a final ginsenoside metabolite produced only through microbial fermentation, that exerts potent anti-inflammatory and AKT-related hypoglycemic activities [39,40,41]. Other rare ginsenosides, Rh2 and Rg3, have also been reported to exhibit anti-diabetic and anti-inflammatory properties by regulating hepatic AKT-mediated insulin signaling and autophagy, respectively [42,43]. In line with these findings, our work provides evidence that FRG shows synergistic effects from multiple fermentation-generated rare ginsenosides, contributing to the management of diabetic conditions during aging. However, further studies are needed to delineate the precise molecular interactions and to determine whether these effects translate to other tissues and biological pathways affected by aging.

As a complex and sophisticated ecosystem, the gut microbiome plays an essential role in host metabolism through bidirectional communication with the liver, facilitating metabolite exchange and modulating immune responses [44,45]. Gut dysbiosis, a state of microbial imbalance, has recently been recognized as one of the hallmarks of aging, contributing to chronic inflammation and increased susceptibility to T2D, often accompanied by an increase in the F/B ratio [7,46]. Thus, modulating the gut microbiota may serve as a therapeutic approach for age-associated diabetes and inflammation [47]. *Erysipelotrichaceae* is one of the families associated with metabolic disorders, exhibiting its elevated abundance in obese or/and diabetic models [48]. *Dubosiella*, a genus within *Erysipelotrichaceae*, has similarly been reported to increase under diabetic conditions [49,50]. In contrast, the genus *Borkfalkiaceae* (also known as *Christensenellaceae*) is considered a commensal group with immunomodulatory effects and protective properties against metabolic disorders [51]. In support of these findings, our microbial compositional and LEfSe analyses revealed that FRG supplementation decreased the F/B ratio and inhibited the increase in *Erysipelotrichaceae* and *Dubosiella*, while elevating the abundance of *Borkfalkiaceae*. We also found that FRG augmented the abundance of the *Clostridia vadin BB60 group* and *Parasutterella* at the genus level. These genera are negatively correlated with metabolic dysfunction, and their restoration through dietary interventions has been shown to ameliorate T2D and inflammation [52,53,54,55]. In the meantime, FRG supplementation increased the abundance of *Ruminococcaceae* (also known as *Oscillospiraceae*) *bacterium* and *Clostridium leptum*, butyrate-producing taxa reported for their decreased abundance under diabetic conditions along with anti-inflammatory properties [56,57,58,59]. This might be due to the prebiotic-like effects of ginsenosides of FRG (e.g., compound K), or FRG per se, subsequently improving insulin homeostasis and inflammation [60,61].

While the current study provides meaningful insight into FRG’s anti-aging effects, some limitations should be considered when interpreting our findings. First, we used male mice to consider the susceptibility to diabetes and exclude the bias from hormonal changes [62], but we cannot rule out whether FRG could exert sexual dimorphism during aging. Second, we set a single dosage (300 mg/kg) of FRG in naturally aged mice over a 14-week intervention period. Even though we carefully determined this dosage and duration based on our previous work demonstrating the safety of RG supplementation in 19-month-old mice [16], it remains unclear whether FRG would exhibit similar anti-aging properties under different conditions, such as lower/higher doses or interventions longer than 4 months. Additionally, more detailed assessments of microbial metabolites and intestinal barrier integrity could provide a more comprehensive understanding of the mechanisms underlying FRG’s benefits on gut health. The contents of short-chain fatty acids and bile acids, and the expression of tight junction proteins in the liver and the gut may serve as informative markers to further support our observations.

## 5. Conclusions

Our study highlights the multifaceted potential of FRG supplementation in mitigating age-associated phenotypic and metabolic changes. FRG attenuated age-related dysregulation of insulin homeostasis by enhancing hepatic AKT and PRAS40 activations and suppressing pro-inflammatory cytokines. These metabolic benefits were accompanied by favorable modifications in gut microbiome structure and composition in naturally aged mice. By decreasing the F/B ratio, FRG reduced the relative abundance of diabetes-related opportunistic taxa while enriching beneficial bacteria, leading to the mitigation of age-associated gut dysbiosis. In conclusion, FRG can be a promising anti-aging functional food candidate to protect against age-driven disruption of insulin homeostasis, hepatic inflammation, and gut microbiome balance.

## Data Availability

The raw reads for liver RNA-seq and 16S rRNA sequencing are openly available in the National Center for Biotechnology Information (NCBI) Sequence Read Archive (SRA) database under the BioProject accession number PRJNA1371365.

## Supplementary Materials

The following supporting information can be downloaded at: https://www.mdpi.com/article/10.3390/biology15030211/s1, Figure S1: Original immunoblots; Table S1: Formulation of FRG-customized animal diet; Table S2: Oligonucleotides for real-time RT-PCR used in the current study; Table S3: Composition profile of FRG by HPLC analysis. Reference [63] is cited in the supplementary materials.
