# Supplementary material for: Fermented Red Ginseng Restores Age-Associated Insulin Homeostasis and Gut Microbiome Balance in Mice
Source: Biology (Basel). 2026 Jan 23;15(3):211. doi: 10.3390/biology15030211 (PMC12896615; doi:10.3390/biology15030211)
Supplement: Supplementary file 1 [file biology-15-00211-s001.zip › biology-4045810-table.pdf]

Table S1. Formulation of FRG-customized animal diet.

Ingredients (g)	NCD (g/kg)	NCD+FRG (g/kg)
**γ-cyclodextrin**	4	0
**Fermented red ginseng (FRG)**[2]	0	4
**Carbohydrates**		
Cornstarch	394.562	394.562
Dyetrose	132	132
Sucrose	90	90
**Protein**		
High nitrogen casein	200	200
**Fat**		
Soybean oil	70	70
**Fiber**		
Cellulose	50	50
Mineral Mix[3]	35	35
Vitamin Mix[3]	10	10
Supplement[3]	10	10
Choline chloride	1.4	1.4
Ethoxyquin	0.024	0.024
L-Cystine	3	3
t-Butylhydroquinone	0.014	0.014

NCD, normal chow diet.

[1] Based on a standard rodent diet (Reeves *et al.*, 1993, *J Nutr*, 123(11), 1939-1951).

[2] Provided by BTC Corporation (Gwacheon-si, Republic of Korea).

[3] Dyets Inc. (Bethlehem, PA, USA) (Catalog #210025, #310025, and #410750 for mineral, vitamin, and supplement mix, respectively).

Table S2. Oligonucleotides for real-time RT-PCR used in the current study.

Origin	Target	Forward (5’–3’)	Reverse (5’–3’)	Gene ID
Mouse (*Mus musculus*)	TNF-α	5’-AGGAGGAGTCTGCGAAGAAGA-3’	5’-GGCAGTGGACCATCTAACTCG-3’	21926
	IL-1β	5’-TGCCACCTTTTGACAGTGATG-3’	5’-TGATGTGCTGCTGCGAGATT-3’	16176
	IL-6	5’-CCATAGCTACCTGGAGTACATG-3’	5’-TGGAAATTGGGGTAGGAAGGAC-3’	16193
	Myd88	5’-GATGACCCCCTAGGACAAACG-3’	5’-ACTCGATATCGTTGGGGCAG-3’	17874
	Cxcl2	5’-CCCAGACAGAAGTCATAGCCAC-3’	5’-TGGTTCTTCCGTTGAGGGAC-3’	20310
	Pai-1	5’-GACACCCTCAGCATGTTCATC-3’	5’-AGGGTTGCACTAAACATGTCAG-3’	18787
	Mcp1	5’-GCATCCACGTGTTGGCTCA-3’	5’-CTCCAGCCTACTCATTGGGATCA-3’	20296
	Mmp12	5’-TGCACTCTGAAAGGAGTCT-3’	5’-GTCATTGGAATTCTGTGGTTTCCA-3’	17381
	TIMP1	5’-CCTTGCAAACTGGAGAGTGACA-3’	5’-AGGCAAAGTGATCGCTCTGGT-3’	21857
	18S	5’-AGTCCCTGCCCTTTCTACACA-3’	5’-CGATCCGAGGGCCTCACTA-3’	19791

Table S3. Composition profile of FRG by HPLC analysis.

	Concentration (μg/mL)	
**Ginsenosides**	**Control***	**FRG**
Compound K	0.0	18.4
Rh2	19.5	156.1
Rh1	27.2	110.7
Rg5+Rk1	210.8	1961.5
Rg2	204.1	580.6
Rg3	86.2	884.9
Rg1	348.9	212.6
Rf	249.1	329.6
Re	1130.9	772.5
Rd	704.5	857.5
Rb2+Rc	2100.4	2354.1
Rb1	1549.9	1683.9

* Non-fermented red ginseng.
