# Supplementary figures and images for: Fermented Red Ginseng Restores Age-Associated Insulin Homeostasis and Gut Microbiome Balance in Mice
Source: Biology (Basel). 2026 Jan 23;15(3):211. doi: 10.3390/biology15030211 (PMC12896615; doi:10.3390/biology15030211)

Figure S1.

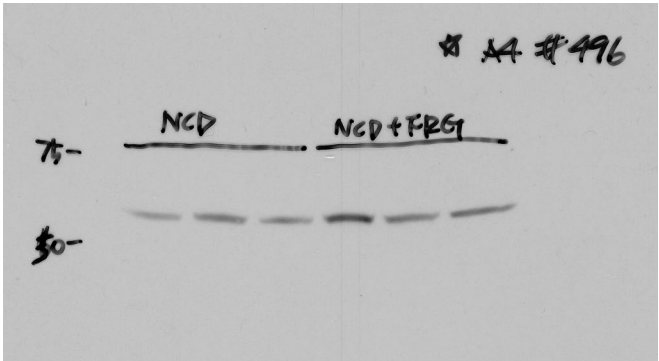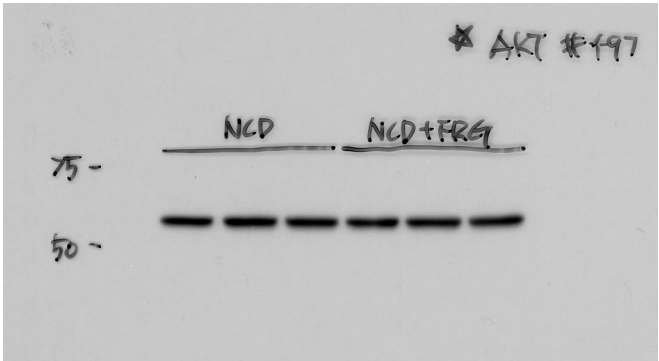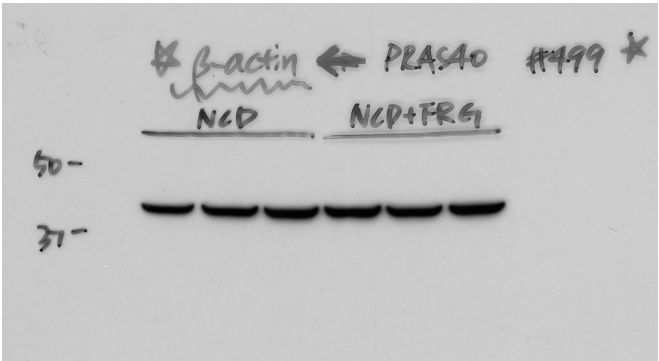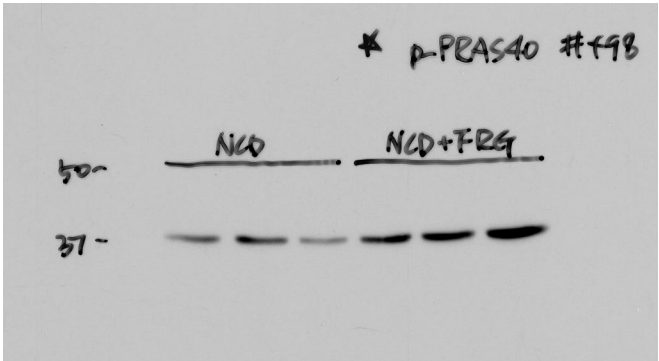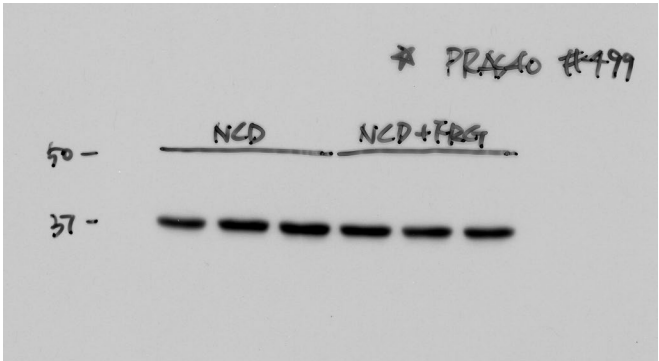

Figure S1.

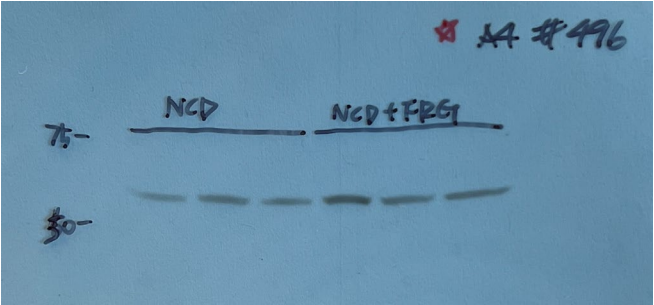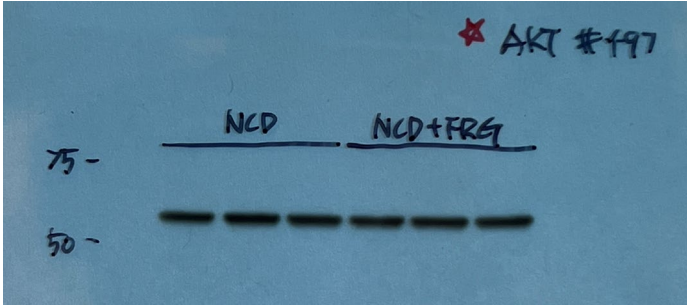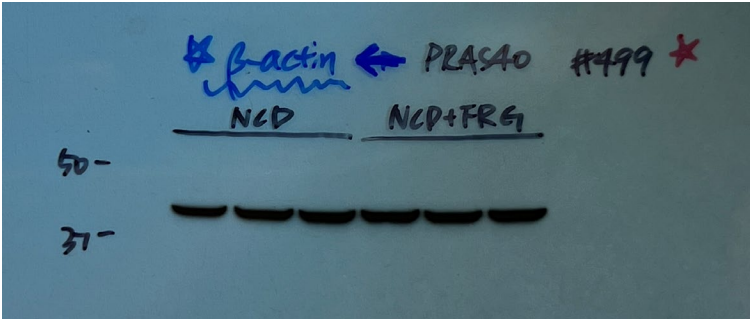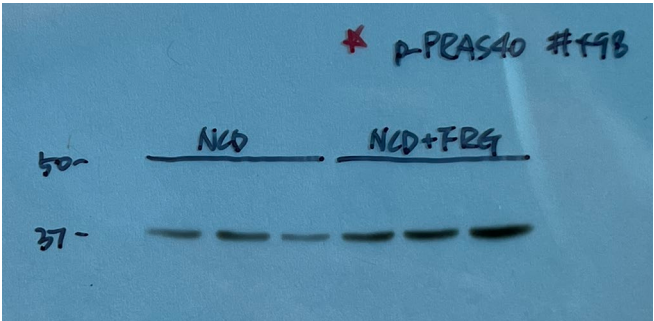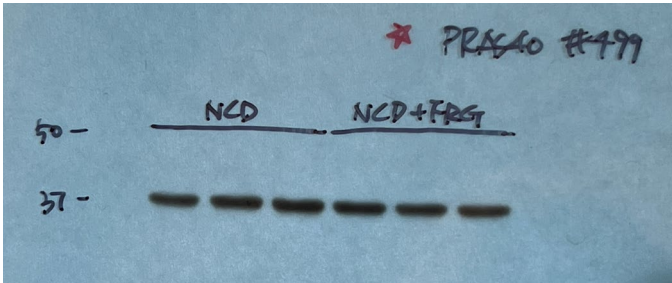

Supplement: Supplementary file 1 [file biology-15-00211-s001.zip › biology-4045810-original-images.pdf]
